# Crayon ingestion

**DOI:** 10.1002/ams2.70030

**Published:** 2025-01-09

**Authors:** Kisho Noda, Tomoki Wada, Ryohei Horie, Toshifumi Asada, Ryota Inokuchi, Kent Doi

**Affiliations:** ^1^ Department of Emergency and Critical Care Medicine The University of Tokyo Hospital Bunkyo‐ku Tokyo Japan

A 24‐year‐old male with autism presented to the emergency department with recurrent vomiting. His mother found crayon fragments in the vomitus. Computed tomography revealed multiple high‐density cylindrical objects in the lower esophagus, stomach, and isolated in the ileum (Figure 1A,B). Urgent upper gastrointestinal endoscopy was performed under general anesthesia and 24 crayon fragments were successfully retrieved from the stomach (Figure 1C,D). The crayons in the ileum were defecated without complications.

**FIGURE 1:**
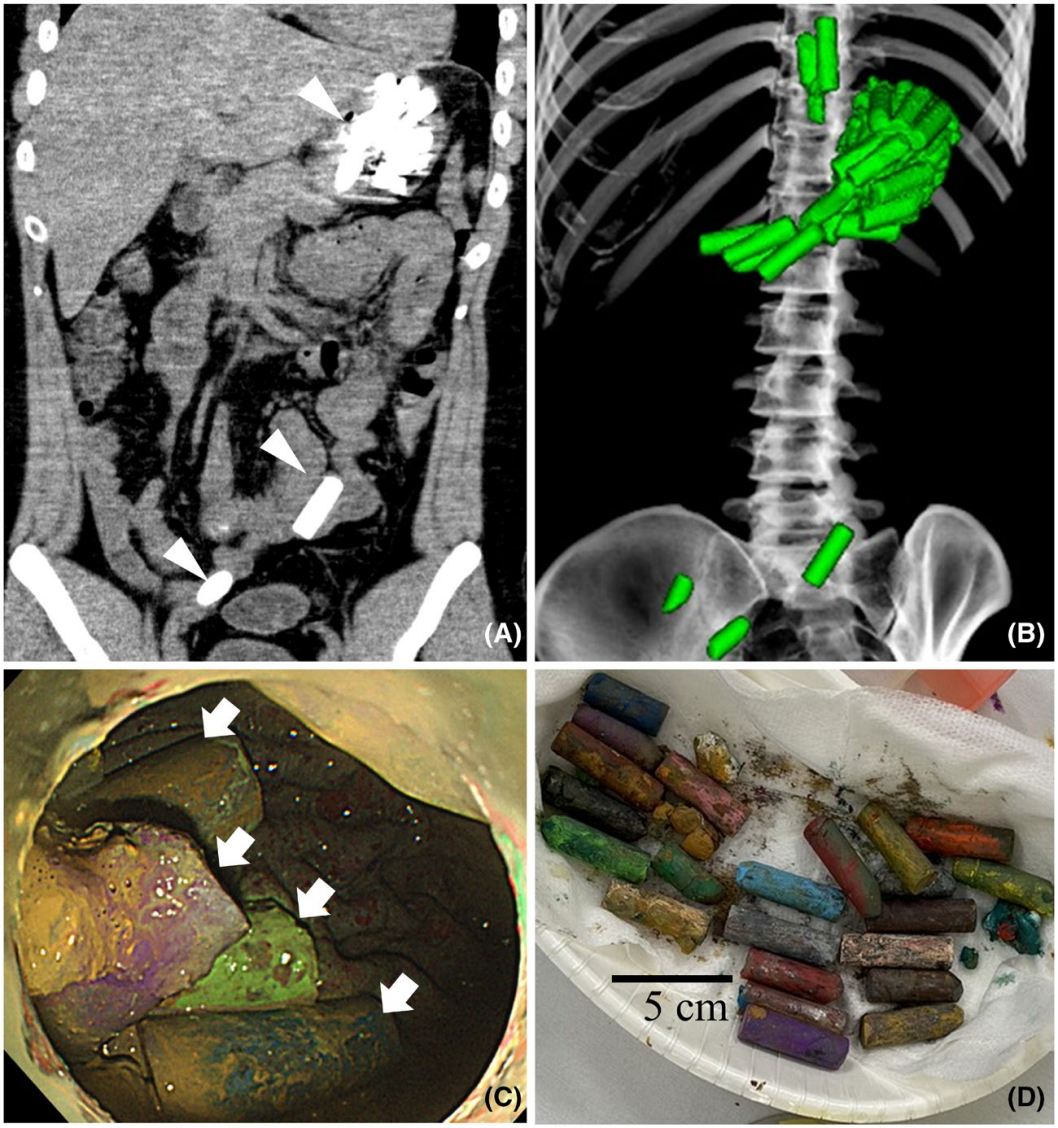
(A) Computed tomography showed multiple high‐density cylindrical objects in the lower esophagus, stomach, and isolated in the ileum (arrow heads). (B) Three‐dimensional (3D) image. (C) Urgent upper gastrointestinal endoscopy revealed crayons in the stomach (arrows). (D) The removed crayons.

Although methemoglobinemia induced by colored crayons has been reported previously,^1^, ^2^ current crayon formulations consist largely of nontoxic wax with an extremely low risk of acute toxicity. Because crayons are not readily digestible enzymatically or thermally in the gastrointestinal tract owing to a melting point of approximately 60°C, they can readily cause complications such as gastrointestinal obstruction or choking on aspirated vomit. Guidelines for the management of foreign bodies in the stomach recommend urgent removal of sharp or long (≧6 cm in length) objects by urgent endoscopy.^3^ In this case, the crayon fragments were blunt and <6 cm each, but urgent endoscopy under general anesthesia was performed because the patient with autism was concerned about being unable to express discomfort or pain when complications occurred.

## CONFLICT OF INTEREST STATEMENT

The authors declare no conflicts of interest.

## ETHICS STATEMENT

Approval of the research protocol: N/A.

Informed consent: Written informed consent was obtained from the patient's family for the publication of this case report and accompanying images.

Registry and registration no. of the study/trial: N/A.

Animal studies: N/A.

## Data Availability

The data that support the findings of this study are available from the corresponding author upon reasonable request.
